# Fewer Doctors Turned out

**Published:** 1916-03

**Authors:** 


### Fewer Doctors Turned Out.

There are only half as many men and women studying to be doctors now as there were twelve years ago, according to Dr. Ross Patterson, who keeps a sensitive finger upon the pulse of American medical schools.

"The decrease in the number of medical students in the United States is extraordinary," says Dr. Patterson. "Twelve years ago there were twenty-nine thousand studying medicine and surgery. Now there are only fifteen thousand."

Evidently American young men and women are keeping away from drugs and the scalpel. But why? Dr. Patterson contends that all big cities have far too many doctors, but that many rural communities have too few.

That these figures are accurate, there is no doubt, but why there is this falling off in the number of medical students there is no satisfactory explanation. It would seem that in this day and time when medical science is beginning to so specialize that almost every part of the body has its particular practioner, and when there are apparently as many ailing people per thousand, notwithstanding all the advance made in sanitation, disinfectants, etc., that more doctors would be needed to supply the demand.

In olden times—yes, in times not so very olden—the average doctor would treat anything from corns to insanity. Now there is the eye, ear and nose specialist; the nerve specialist; the specialist in gynecology; the baby specialist, and this and that specialist, and it would seem that the ranks of the medical men would have to be greatly increased to cover all the divided field.

Still, if the medical colleges are turning out fledgling doctors at the rate of 15,000 per year, we need not begin to worry yet awhile about a scarcity, especially as a doctor is proverbially long lived, and there are many coming on, with but few of them leaving us.

